# Overexpression of phosphoserine aminotransferase PSAT1 stimulates cell growth and increases chemoresistance of colon cancer cells

**DOI:** 10.1186/1476-4598-7-14

**Published:** 2008-01-25

**Authors:** Nadia Vié, Virginie Copois, Caroline Bascoul-Mollevi, Vincent Denis, Nicole Bec, Bruno Robert, Caroline Fraslon, Emmanuel Conseiller, Franck Molina, Christian Larroque, Pierre Martineau, Maguy Del Rio, Céline Gongora

**Affiliations:** 1 CNRS, UMR 5160, CRLC, 15, av, Charles Flahault, BP14491, 34093, Montpellier Cedex 5, France; 2 INSERM, EMI229, Centre Régional de Lutte contre le Cancer Val D'Aurelle-Paul Lamarque, Montpellier, France; 3 Unité de Biostatistique, Centre Régional de Lutte contre le Cancer Val D'Aurelle-Paul Lamarque, Montpellier, France; 4 INSERM, U860, Centre Régional de Lutte contre le Cancer Val D'Aurelle-Paul Lamarque, Montpellier, France; 5 Service d'Oncologie Digestive and Département d'Oncologie, sanofi aventis, Vitry-sur-Seine, France; 6 Univ Montpellier I, IRCM, Montpellier, France

## Abstract

**Background:**

Colorectal cancer (CRC) is one of the most common causes of cancer death throughout the world. In this work our aim was to study the role of the phosphoserine aminotransferase PSAT1 in colorectal cancer development.

**Results:**

We first observed that PSAT1 is overexpressed in colon tumors. In addition, we showed that after drug treatment, PSAT1 expression level in hepatic metastases increased in non responder and decreased in responder patients.

In experiments using human cell lines, we showed that ectopic PSAT1 overexpression in colon carcinoma SW480 cell line resulted in an increase in its growth rate and survival. In addition, SW480-PSAT1 cells presented a higher tumorigenic potential than SW480 control cells in xenografted mice. Moreover, the SW480-PSAT1 cell line was more resistant to oxaliplatin treatment than the non-transfected SW480 cell line. This resistance resulted from a decrease in the apoptotic response and in the mitotic catastrophes induced by the drug treatment.

**Conclusion:**

These results show that an enzyme playing a role in the L-serine biosynthesis could be implicated in colon cancer progression and chemoresistance and indicate that PSAT1 represents a new interesting target for CRC therapy.

## Background

Colorectal cancer (CRC) is the fourth cause of cancer-related deaths worldwide and 945 000 new cases are detected every year. Fifteen percent of CRC are hereditary, whereas the other 85% are considered as being sporadic. CRC development involves multiple genetic events like genetic mutations and aberrant gene expression that have been well documented by Vogelstein and colleagues [1]. These authors proposed a multistep model leading to colorectal tumorigenesis. One of the first genetic mutations in CRC development is a mutation in the APC/β-catenin pathway, resulting in adenoma formation. Then, a mutation in another growth pathway, either Ki-Ras/BRAF, Smad4/TGFβ, PI3K, or TP53/BAX occurs when the adenoma becomes larger, leading to carcinoma. Besides this well-characterized model, it appears that a large number of genes (mainly identified by microarray) could present an altered expression pattern and may play a role in initiation, progression, and drug response of CRC [2].

CRC primary treatment is surgery but the risk of recurrence due to undetected micrometastases is high. To reduce this risk, chemotherapy can be administrated after removal of the primary tumor in the advanced stages. Combination of the antimetabolite 5-fluorouracil (5-FU) and folinic acid with the topoisomerase I inhibitor irinotecan and/or the platinum compound oxaliplatin appears to be the most effective treatment, with a response rate between 40 and 50% [3,4]. Recently, new biological therapeutic agents like the monoclonal antibodies cetuximab and bevacizumab have emerged and improved the clinical outcome of patients with colorectal metastatic cancer [5,6]. However, since 50% of patients are still not responsive to chemotherapy because of drug resistance, prediction of patient response and development of alternative treatments is of prime importance in the CRC field. Using genome-wide transcriptional analysis, several authors have identified new predictive markers and therapeutic targets implicated in the development and drug response of colon carcinoma cells [7-10].

The phosphoserine aminotransferase PSAT1 is an enzyme implicated in serine biosynthesis and has been linked with cell proliferation in vitro [11]. Two studies reported that PSAT1 mRNA is overexpressed in colon adenocarcinoma [12] and increases with tumor stage in colon cancer [13]. It was also shown that high PSAT1 mRNA levels in breast cancer are associated with a poor clinical response to endocrine therapy [14]. These studies showed that there is a clear rational for studying PSAT1 as a pro-proliferative and pro-survival factor in the context of colon cancer.

This report addresses the role of the phosphoserine aminotransferase PSAT1 in CRC. We first observed that PSAT1 was overexpressed in tumor samples from CRC patients, and that its level of overexpression after chemotherapy is correlated with poor regression of the tumour metastases. Then, we demonstrated that PSAT1 ectopic expression promoted cell growth and made the cells more resistant to oxaliplatin treatment. Overall, the data presented here identify PSAT1 as a potential new therapeutic target in CRC.

## Results

### PSAT1 is overexpressed in colorectal tumor samples

**Figure 1:**
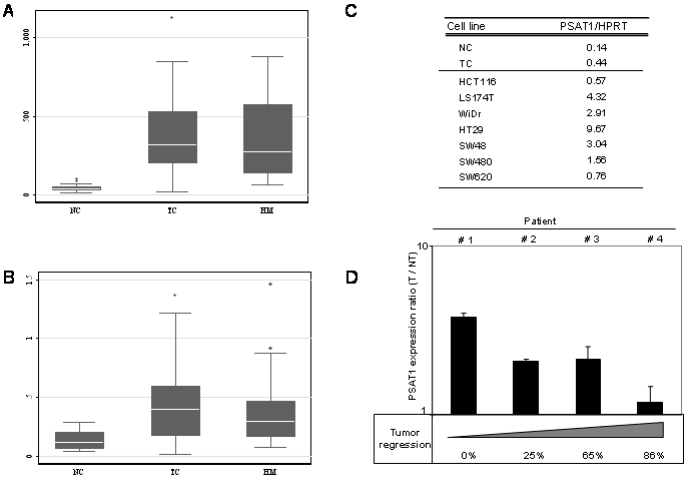
**PSAT1 mRNA expression level and clinical correlation between PSAT1 mRNA expression level and tumoral regression**. Box plot representation of PSAT1 mRNA levels determined by Affymetrix technology (A) and by semiquantitative PCR (B). NC: normal colon (17 samples). TC: tumor colon (23 samples). HM: hepatic metastasis (22 samples). The box for each gene represents the interquartile range (25–75th percentile) and the line within this box is the median value. Bottom and top bars of the whisker indicate the 10th and 90th percentiles, respectively. Outlier values are indicated (closed squares). The clinical data for the 29 patients included in this study are detailed in Table 1. C, PSAT1 mRNA levels determined by semiquantitative PCR in various cell lines. NC: normal colon (17 samples). TC: tumor colon (23 samples). D, PSAT1 mRNA expression in hepatic metastasis before (NT: not treated) and after (T: treated) chemotherapy determined by semi-quantitative RT-PCR. Patients' response to treatment (tumor regression) and PSAT1 expression after therapy are negatively correlated (Pearson's test, p = -0.76).

To investigate the role of PSAT1 in colorectal cancer, we first analyzed the PSAT1 mRNA expression in tumors from 29 patients with advanced colorectal cancer. In Fig. 1 are shown the expression levels of PSAT1 measured both by Affymetrix (Fig. 1A) and Q-PCR (Fig. 1B) experiments in 23 colon tumors (TC), 22 hepatic metastases (HM) and 17 normal mucosas (NC). Using both methods, we found that the phosphoserine aminotransferase PSAT1, was significantly overexpressed in tumors (NC *vs *TC, p < 0.0001 and NC *vs *HM, p < 0.0001). The fold change was less important when measured by Q-PCR than by microarray (3.0 compared to 7.9 for TC/NC and 2.9 compared to 7.2 for HM/NC). This can be explained by the difference in sensitivity of each technique or by the variation of the microarray data which were performed only once for each sample, whereas the Q-PCR measurements were done in triplicates.

**Table 1:** Clinical data for patient samples.

		**Number**	**Percentage**
**Gender**			
	Masculine	18	62.1%
	Feminine	11	37.9%
**Age**			
	Median	60.2	
	Range	(45.1: 76.0)	
**Tumor Location**			
	Colon Right	1	3.4%
	Colon transverse	2	6.9%
	Colon Left	20	69.0%
	Rectum	3	10.3%
	Recto-sigmoid	3	10.3%
**Histological Type**			
	ADK well diff	11	37.9%
	ADK diff	11	37.9%
	ADK not well diff	3	10.3%
	ADK (diff)	3	10.3%
	Missing	1	
**N**			
	Nx	1	3.4%
	N0	7	24.1%
	N1	5	17.2%
	N2	16	55.2%
**T**			
	Tx	1	3.4%
	T3	19	65.5%
	T4	9	31.0%
**OMS initial**			
	0	10	45.5%
	1	12	54.5%
	missing	7	

Abbreviation: ADK, adenocarcinoma, N, node, T, tumor.

We then investigate the PSAT1 expression level in colon cancer cell lines. Semiquantitative RT-PCR analysis of PSAT1 mRNA level in HT29, SW620, SW480, SW48, LS174T and WiDR cell lines was performed. mRNA level in normal and tumoral colon from samples showed in figure 1B were also included as an example of non-cancerous and cancerous intestinal epithelial cells respectively (Fig. 1C). PSAT1 was expressed in all the colon cancer cells tested, with the lowest expression level found in normal colon.

### Increase of PSAT1 expression after chemotherapy is linked with tumor progression in CRC patients

To determine whether the high PSAT1 mRNA levels is associated with clinical response, we explored the correlation between PSAT1 expression levels and tumor regression after chemotherapy. Microarray and RT-Q-PCR analyses were performed with mRNA extracted from hepatic metastases from four patients with metastatic colon cancer before and after FOLFIRI treatment (leucovorin, 5-FU and irinotecan). We defined the percentage of regression for each patient on the basis of anatomic indicators (tumor lesions) according to WHO criteria. Among the four patients, one was highly sensitive to FOLFIRI, with a tumoral regression close to 85%; two were partially sensitive, with tumoral regression ranging from 65% to 25%; and one was considered as resistant, with no measurable tumoral regression. The PSAT1 expression ratio (ratio of the mRNA level in hepatic metastases after to before FOLFIRI treatment) for each patient has been calculated using Affymetrix microarrays (data not shown) and RT-Q-PCR values (Fig. 1D). For the patient showing the best tumor regression (patient 4), the PSAT1 ratio was very low. For patients showing mild tumor regression (patients 2 and 3), the PSAT1 expression ratio increased, and this increase was greater when tumor regression was poor. Finally, the patient showing no tumor regression (patient 1) had a high PSAT1 ratio. This inverse correlation between clinical progression and the PSAT1 ratio in tumor specimens was obtained using both RNA quantification methods, Affymetrix microarrays (data not shown) and RT-Q-PCR (Fig. 1D). This suggests that an increase in PSAT1 overexpression after chemotherapy may be correlated with a poor treatment response.

### SW480-PSAT1 colon carcinoma cells present an advantage in proliferation and survival in low serum conditions

**Figure 2:**
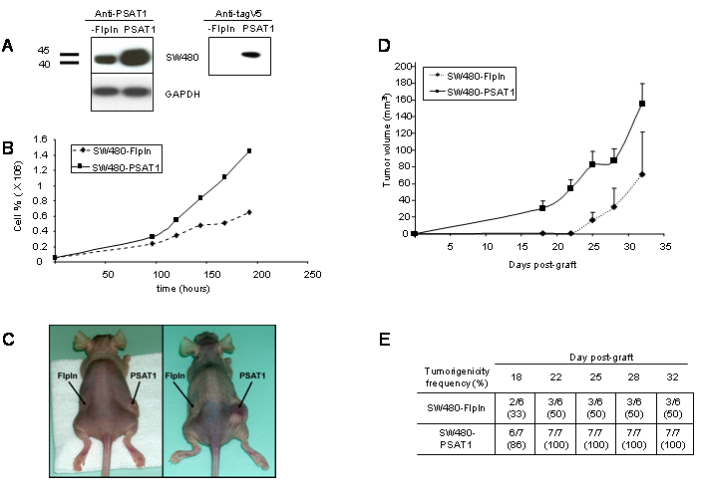
**Effect of PSAT1 overexpression on cell proliferation and tumor xenograft**. A, Western blot analysis of PSAT1 expression in SW480-FlpIn and SW480-PSAT1 cell lines using anti-PSAT1 or anti-V5 antibodies. B, Growth curve of SW480-FlpIn and SW480-PSAT1 cultured in low serum medium (1%). The curves are representative of three experiments. C, Representative nude mice, SW480-FlpIn were injected subcutaneously into the left flank and SW480-PSAT1 into the right flank. D, Tumor volume evolution after a subcutaneous xenograft of SW480-FlpIn and SW480-PSAT1 in 7 athymic nude mice. E, In vivo tumorigenic frequency for SW480-FlpIn and SW480-PSAT cell lines. Ratios indicate the number of mice presenting an observable tumor compared with the total number of mice observed. Numbers in parentheses indicate percentages.

To determine the PSAT1 overexpression effect in human adenocarcinoma cells, we stably transfected PSAT1 in SW480 using the Flp-In System. In this system, the recipient cell line contains a single integrated Flp recombination site where the ectopically expressed gene can be inserted. This system produces isogenic cell lines in which all the transfected clones produce equivalent levels of the protein of interest, thereby eliminating the need to analyze multiple subclones. As a control, we used the SW480-FlpIn cell line, stably transfected with the empty vector to avoid the possibility of observing a phenotype caused only by an integration effect. PSAT1 was detected by immunoblotting using an anti-PSAT antibody. Fig. 2A shows that PSAT1 was endogenously expressed in the control SW480-FlpIn cells with an apparent molecular weight of 40 000. In the stable SW480-PSAT1 clone, the ectopic PSAT1 protein displayed a higher molecular weight of 45 000. This higher molecular weight for the transfected protein is due to the presence of the C-terminal V5 tag in our construct. Two forms of human phosphoserine aminotransferase have been reported, PSAT1α and PSAT1β, encoding, respectively, a protein of 35 200 and 40 000. Fig. 2A shows that the SW480-FlpIn and SW480-PSAT1 cells only expressed the PSAT1β form, which has been reported to be the physiologically functional enzyme.

We first analyzed the growth rates of the SW480-FlpIn and SW480-PSAT1 cells. The proliferation assay showed that SW480-PSAT1 grew faster than SW480-FlpIn in standard culture conditions (10% of FBS), with an average doubling time of 22.05 ± 1.58 hours and 30.54 ± 3.88 hours, respectively (data not shown). Thus, expression of PSAT1 in the SW480 cells conferred a proliferative advantage since the doubling time decreased by about eight hours. The same results were obtained in HEK293 epithelial kidney cells (data not shown), indicating that this effect is not cell line dependent. In low-serum medium (1% of FBS), proliferation of both cell lines decreased but SW480-PSAT1 grew still faster than SW480-FlpIn, with an average doubling time of 36.3 ± 5.9 hours and 53.5 ± 6.5 hours, respectively (Fig. 2B). SW480-PSAT1 growth rate in low-serum conditions is comparable with the SW480-FlpIn's in 10% FBS (36.3 hours compared to 30.54 hours, respectively) indicating that SW480-PSAT1 showed partial growth factor independence, which is a hallmark of malignancy.

### SW480-PSAT1 colon carcinoma cells present a high tumorigenic potential in tumor xenografts

To evaluate the tumorigenic potential of SW480-FlpIn and SW480-PSAT1, we compared the ability of both cell lines to give rise to subcutaneous tumors in nude mice. Cells were injected subcutaneously into the left flank for SW480-FlpIn and the right flank for SW480-PSAT1 of the same nude mouse and tumor growth was evaluated by measuring the tumor size at different times. As shown in Fig. 2C and 2D, the SW480-PSAT1 tumor grew much faster than the SW480-FlpIn tumor, indicating that colon cancer cells overexpressing PSAT1 proliferated faster *in vivo*, as was also the case *in vitro*. In addition, 18 days after cell inoculation, 86% of SW480-PSAT1 grafted mice developed a tumor whereas only 33% of SW480-FlpIn grafted mice developed one (Fig. 2E). Moreover, even after 30 days only half of the mice developed a tumor when grafted with SW480-FlpIn whereas all the mice grafted with SW480-PSAT1 developed a tumor after only 22 days.

### SW480 colon carcinoma cells overexpressing PSAT1 are more resistant to drug treatment

**Figure 3:**
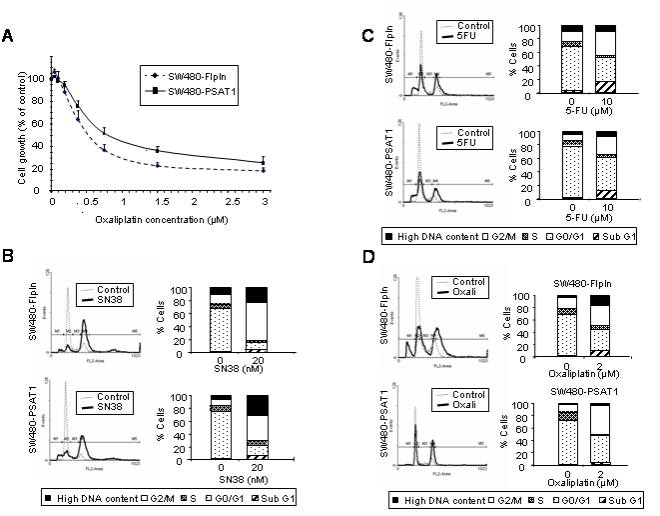
**Effect of PSAT1 overexpression on drug resistance and cell cycle progression**. A, Comparison of growth inhibition (IC_50_) between SW480-FlpIn and SW480-PSAT1. Cell cycle distribution of SW480 and SW480-PSAT1 cells following treatment with 20 nM SN38 (B), 10 μM 5-FU (C) and 2 μM oxaliplatin (D) for 72 hours. Cell cycle distribution was measured by propidium iodide staining with a FACScan flow cytometer. FACS profiles and the proportion of cells in each phase of the cell cycle are presented: cells with a high DNA content (black), cells in G2/M (white), S (grey), GO/G1 (white with black dots) and the subG1 phase (white and black slashes). Cells in subG1 represent cells in late apoptosis.

We further characterized the SW480-FlpIn and SW480-PSAT1 cell lines for their responses to chemotherapy. Both cell lines were treated with the three drugs commonly used in the treatment of colon cancer: 5-FU, SN38 (the active metabolite of irinotecan), and oxaliplatin. The cells were exposed for 72 hours to increasing concentrations of each drug and their growth was measured using the sulforhodamine B assay. When treated with SN38, SW480-PSAT1 cells (IC50 = 10.3 ± 0.57 nM) were only marginally more resistant (p = 0.05) than the control SW480-FlpIn (IC50 = 9 ± 0.11 nM), indicating that PSAT1 is not playing a role in SN38 resistance (data not shown). For 5-FU, the IC50 values of the SW480-FlpIn and SW480-PSAT1 cells were respectively 2.98 ± 0.86 μM and 4.27 ± 0.42 μM (data not shown) but the difference is not significant (p = 0.1), showing that PSAT1 is not implicated in resistance to 5-FU. Finally, we observed that oxaliplatin treated cells displayed IC50 values of 0.54 ± 0.06 μM for SW480-FlpIn and 0.81 ± 0.13 μM for SW480-PSAT1 cells (Fig. 3A) (p = 0.014), and IC70 values were 0.93 ± 0.13 and 2.19 ± 0.51 respectively (Fig. 3A). These results indicate that SW480 cells overexpressing PSAT1 were more resistant to drug, notably oxaliplatin, than the mock transfected cells.

We then compared the drug effect on cell cycle and apoptosis of SW480-FlpIn and SW480-PSAT1 cells, using drugs concentrations corresponding to the IC70 value. Twenty nM of SN38 during 72 hours induced a G2/M arrest with a high number of polyploid cells (up to 38%) for both cell lines (Fig. 3B). Following a 10 μM treatment of 5-FU, both cell lines presented the same increase in the number of cells in the G2/M phase and a diminution in the G0/G1 and S phases (Fig. 3C). These data indicate that SN38 and 5-FU effect on the cell cycle did not depend on PSAT1 expression. Finally, 2 μM oxaliplatin induced a lost of the S phase coupled with an enrichment of the G2/M phase, and this phenotype was enhanced in SW480-PSAT1 cells compared with SW480-FlpIn cells (Fig. 3D). Oxaliplatin induced appearance of polyploid cells in SW480-FlpIn (17%) but not in SW480-PSAT1 (4%). These data indicate that PSAT1 does not seem to be implicated in cell cycle changes after 5-FU and SN38 exposure but rather plays a role in oxaliplatin-induced cell cycle modifications.

Cells in the subG1 phase are representative of late apoptosis. SN38- (Fig. 3B) and 5-FU- (Fig. 3C) induced apoptosis were comparable, with values for SW480-PSAT1 and SW480-FlpIn, respectively, of 7% and 4% for SN38 and 12% and 17% for 5-FU. On the contrary, oxaliplatin induced more apoptosis in SW480-FlpIn (10%) than in the PSAT1 overexpressing SW480 (3%) (Fig. 3D).

### PSAT1 effect on oxaliplatin resistance is mediated by cell death inhibition

**Figure 4:**
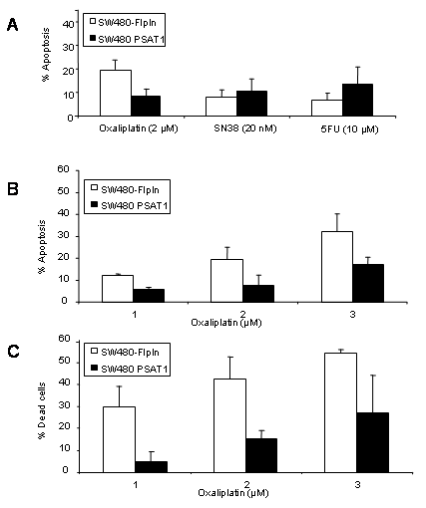
**PSAT1 overexpression effects on cell death after drug treatment**. A, Early apoptosis of SW480 and SW480-PSAT1 cells following exposure to oxaliplatin (2 μM), SN38 (20 nM) and 5-FU (10 μM) for 72 hours. Apoptosis was determined by propidium iodide and Annexin V-FLUOS staining with a FACScan. B, Specific apoptosis of SW480 and SW480-PSAT1 cells following exposure to 1 μM, 2 μM, and 3 μM of oxaliplatin for 72 hours. C, Dead cell number in response to oxaliplatin treatment (1 μM, 2 μM and 3 μM). The number of dead cells was determined by counting the trypan blue stained cells.

Both cell lines were then characterized for drug induced apoptosis by measurement of annexin V-positive and PI-negative cells, representing early apoptosis (compared with late apoptosis, quantified previously by counting the subG1 cells). Cells were treated for 72 hours with either 10 μM of 5-FU, 20 nM of SN38, or 2 μM of oxaliplatin. As shown in Fig. 4A, 5-FU and SN38 induced apoptosis did not vary between SW480-FlpIn and SW480-PSAT1 cell lines, in accordance with the results indicated above on cells in subG1. Combined with the cell cycle data, it appears that SW480-PSAT1 resistance to 5-FU is not due to apoptosis resistance or cell cycle modification but rather to a growth advantage or resistance to apoptosis-independent cell death. Concerning oxaliplatin, SW480-PSAT1 cells displayed less apoptosis (up to two times) than the mock transfected SW480-FlpIn (Fig. 4A), and this may explain in part the resistant phenotype (Fig. 3A).

To characterize more precisely the oxaliplatin induced apoptosis, we used increasing amounts of the drug. SW480-FlpIn and SW480-PSAT1 cells were treated for 72 hours with 1 μM, 2 μM and 3 μM of oxaliplatin. Both cell lines displayed increased apoptosis as the oxaliplatin concentration increased, but SW480-PSAT1 showed for each dose a 50% reduction of the apoptotic response compared with SW480-FlpIn (Fig. 4B). We next investigated the total number of dead cells in response to oxaliplatin treatment to determine if apoptosis was the only mechanism of cell death responsible for this drug toxicity. We counted the trypan blue-labeled dead cells in a cell population treated with oxaliplatin at 1 μM, 2 μM and 3 μM for 72 hours. As shown in Fig. 4C, cell death increased with the oxaliplatin concentration, and the SW480-PSAT1 population exhibited a smaller number of dead cells than the control SW480-FlpIn population, as was also the case when apoptosis was monitored (Fig. 4B). In addition, the total number of dead cells was greater than the number of apoptotic cells, and the difference between the two cell lines was also higher than what we observed with apoptosis. This suggests that PSAT1 overexpression inhibited not only the apoptotic response but also another cell death mechanism induced by oxaliplatin treatment. Besides apoptosis, it appears that mitotic catastrophe, autophagy, and necrosis can participate in tumor cell death [15], indicating that PSAT1 may play an inhibitory role in one of these biological processes. The observation that SW480-FlpIn cells treated with oxaliplatin displayed G2/M arrest and appearance of polyploid cells (Fig. 3D) prompted us to analyze the two phenomena in more detail.

**Figure 5:**
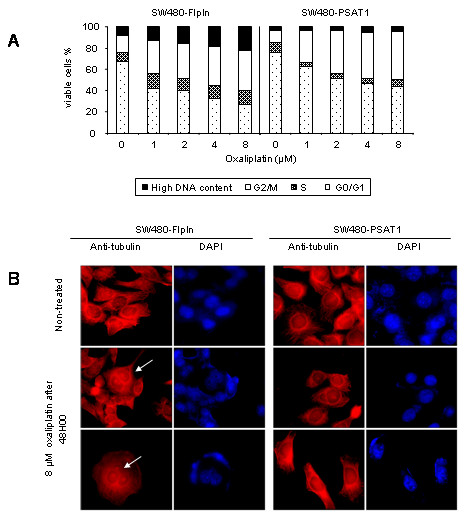
**Cell death resistance in response to oxaliplatin treatment**. A, Proportion of viable cells after a 72 hour oxaliplatin treatment (0 to 8 μM) of SW480-FlpIn and SW480-PSAT1 cells with a high DNA content (black), in the G2/M phase (white), the S phase (grey), or the GO/G1 phase (white with black dots). B, Pictures of SW480-FlpIn and SW480-PSAT1 cell morphology. Cells were either not treated or treated with 10 μM of oxaliplatin for 24 hours. Staining was done using either DAPI to visualize the nucleus or an anti-tubulin antibody to visualize the entire cell. Giant multinucleated cells are indicated by an arrow. Magnification ×63.

To this end, SW480-FlpIn and SW480-PSAT1 cells were treated with increasing concentrations of oxaliplatin (from 0 to 8 μM) during 48 hours, and the cell cycle was monitored by flow cytometry. The analysis presented in Fig. 5A shows an increase in G2/M arrest with increasing concentrations of oxaliplatin in SW480-FlpIn and SW480-PSAT1 and polyploidy formation only in the SW480-FlpIn cells. The number of cells with a DNA content greater than 4N was much lower in SW480-PSAT1 (4% at 8 μM) than in SW480-FlpIn (22% at 8 μM). Furthermore, observation of cell morphology showed the appearance of huge multinucleated cells with unusual shapes consequent to oxaliplatin treatment (Fig. 5B). These cells are similar to the multinucleate giant cells previously reported in curcumin-treated cells [16]. Such a morphological feature along with appearance of high DNA content is compatible with death through mitotic catastrophe, a form of non-apoptotic cell death caused by aberrant mitosis.

### SW480 cells overexpressing PSAT1 are able to recover from a treatment with a high dose of oxaliplatin

**Figure 6:**
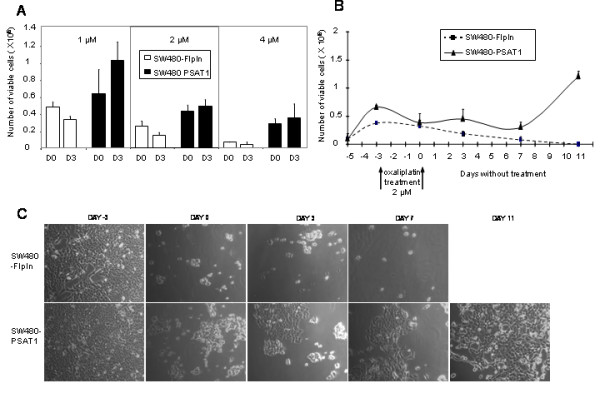
**Cell survival after high dose oxaliplatin treatment and release**. A, Number of viable cells determined after oxaliplatin treatment (1 μM, 2 μM, and 4 μM) of SW480 and SW480-PSAT1 cells. Oxaliplatin treatment lasted for three days and the first counting was done at the end of the three days treatment (D0). Cells were then washed and incubated in complete medium without oxaliplatin (release). Second counting was done three days after the oxaliplatin release (D3). Number of viable cells was determined by counting, excluding trypan blue stained cells. Data are the mean of three independent experiments. B, Cell survival analysis during the three days of oxaliplatin treatment (2 μM) followed by 11 days without drug. The number of viable cells was determined by counting, excluding trypan blue stained cells. Data are the mean of three independent experiments. C, Images of SW480-FlpIn and SW480-PSAT1 cell morphology. Day -3 corresponds to the first day of oxaliplatin treatment (2 μM). Day 0 corresponds to the last day of oxaliplatin treatment. Days 3, 7, and 11 correspond to the number of days after the oxaliplatin release. Magnification ×10.

To investigate the long-term implications of the reduced cell death and growth inhibition in response to oxaliplatin, we evaluated the ability of the cells to grow after a high dose treatment. The cells were exposed to oxaliplatin for 72 hours, then washed and resuspended in growth medium. We counted viable cells at the end of the oxaliplatin treatment (Fig. 6A, D0) and 3 days after drug removal (Fig. 6A, D3). For oxaliplatin concentrations of 1 μM and 2 μM, SW480 mock transfected cells failed to recover from treatment, as suggested by the fact that the cell number at D3 was lower than the cell number at D0. This was not the case for the SW480-PSAT1 cells, which were able to grow between D0 and D3 (Fig. 6A). It must be noted that the drug concentrations used in this assay correspond to almost twice that of the IC50 determined by the sulforhodamine assay, namely, 0.54 μM for SW480-FlpIn and 0.81 μM for SW480-PSAT1, indicating that the difference between cell lines was not simply due to a difference in IC50 and may be called the "long-term drug resistance effect". Indeed, under equitoxic conditions (1 μM of oxaliplatin for SW480-FlpIn and 2 μM for SW480-PSAT1, Fig. 6A), the control cell number continued to decrease after drug removal, whereas SW480 overexpressing PSAT1 continued to grow. Concerning the 4 μM concentration, even though the number of SW480-PSAT1 cells was greater than that of SW480-FlpIn, the SW480-PSAT1 cells were not able to grow after drug removal.

After treatment with 2 μM of oxaliplatin, we followed cell growth for 11 days. As shown in Fig. 6B, the SW480-PSAT1 cells started growing normally 7 days after the end of the treatment, with a doubling time comparable to that for non-treated cell. This was not the case for the control cells which continued to decline after drug removal and were all dead after 7 days, demonstrating that PSAT1 plays a role in cell survival in the presence of oxaliplatin. This was confirmed by observation of the cells by light microscopy. As shown in Fig. 6C, the SW480-PSAT1 cells looked healthier compared with the SW480-FlpIn cells and after 11 days they formed a layer in the dish. In addition, after 11 days, the cell cycle of SW480-PSAT1 returned to normal (data not shown).

Altogether, our data demonstrate that two mechanisms may explain the *in vitro *resistance to oxaliplatin of adenocarcinoma cells overexpressing PSAT1. First, cells are more resistant to oxaliplatin-induced cell death and, second, cells are able to recover from the oxaliplatin induced G2/M arrest.

## Discussion

An important goal in colon cancer therapy is to overcome drug resistance either by deciphering resistance mechanisms or by identifying new drugs with synergistic effects when administered with current treatments. In this study, we observed that the phosphoserine aminotransferase PSAT1 is overexpressed in colon carcinoma compared with normal mucosa. Its overexpression enhances cell proliferation even under low serum condition indicating that PSAT1 may be implicated in cell survival under stress conditions. In addition, we showed that PSAT1 is implicated in drug resistance and, in particular, we demonstrated that the effect of PSAT1 on oxaliplatin resistance is due to cell death inhibition.

PSAT1 belongs to subgroup IV of aminotransferases and catalyses the conversion of 3-phosphohydroxypyruvate to l-phosphoserine. PSAT1 regulates the biosynthesis of serine from intermediates of the glycolytic pathway, and its activity is broadly distributed among the organs but is increased in tissues with a high rate of cell turnover. PSAT1 mRNA is expressed at high levels in the brain, liver, kidney, and pancreas; but at low levels in the thymus, prostate, testis, and colon [11]. Previously, two studies reported that phosphoserine aminotransferase is overexpressed in colon adenocarcinoma [12] and increases with tumor stage in colon cancer [13]. Moreover, Martens and colleagues reported that PSAT1 is overexpressed in some breast cancer samples and that this overexpression is associated with unfavorable clinical outcome in patients treated with the antiestrogen tamoxifen [14]. This last result combined with ours suggests that PSAT1 overexpression is connected to therapy resistance.

It has been reported that treatment of adenocarcinoma colon cells with SN-38, oxaliplatin or 5-FU induces G2/M cell cycle arrest, [16-18], and our data showed that this is also the case in PSAT1-overexpressing cells. However, in the case of oxaliplatin, cell cycle modifications were amplified by PSAT1 overexpression since, in the transfected cell line; more cells were blocked in G2/M phase. In addition, in oxaliplatin-treated SW480 cell cultures; there was an accumulation of large cells with a high DNA content, as seen by FACS analysis and immunofluorescence microscopy. This phenotype is reminiscent of a cell death mechanism called mitotic catastrophe, which results from aberrant mitosis and leads to the formation of large nonviable cells with numerous micronuclei [19]. To date, induction of mitotic catastrophes by oxaliplatin has not been reported, but the fact that oxaliplatin causes DNA strand breaks and G2/M arrest is in good agreement with previously described mechanisms that induce mitotic catastrophe [20]. We propose that oxaliplatin induced cell death in SW480 cells is due to both apoptosis and mitotic catastrophes and that the resistance phenotype of the PSAT1 overexpressing cells results from a decrease in the induction of both mechanisms. This suggests that prevention of mitotic catastrophes could be a new mechanism for drug resistance in colon adenocarcinoma cells.

The study reported herein suggest that PSAT1 may be a gene whose expression pattern before and after treatment is associated with poor response to FOLFIRI in colorectal cancer patients. Our *in vitro *results indicating that PSAT1 overexpression impairs the effect of the chemotherapy fit well with these clinical data. We are aware that data on four clinical samples is not statistically significant and that this result must be confirmed on more samples. We are planning to collect such metastatic samples taken from the same patient before and after drug therapy, especially patients treated with FOLFOX (leucovorin, 5-FU and oxaliplatin).

PSAT1 is implicated in catabolism, catalyzing the second step in the biosynthesis of the amino acid serine [21]. The serine synthesis pathway provides the precursors for cellular proliferation as it is required for the synthesis of purine nucleotides. Knowing that PSAT1 is implicated in purine nucleotide synthesis, one drug that could overcome chemoresistance due to PSAT1 overexpression may be the antimetabolite purine antagonist mercaptopurine (thioguanine), which inhibits several enzymes in the purine synthesis pathway. This family of compounds is widely used for autoimmune disorder and for patients with chronic inflammatory bowel diseases [22].

It is interesting to note that among all the amino acids, serine has an important role in cell proliferation [23]. This was demonstrated for the first time in 1956 when McCoy demonstrated that cells in culture grew better when serine and glycine were added to the culture medium [24]. In addition, it was shown by Snell [25] that 3PGHD activity (enzyme that catalyses the initiating step in serine biosynthesis) is increased in human colon carcinoma. These two reports along with our data indicate that the L-serine synthesis pathway may be important in colon tumor formation and growth and deserves further investigations.

## Conclusion

In summary, our results point to PSAT1 as a novel gene that stimulates colorectal cancer cells proliferation and modulates chemotherapy sensitivity, both *in vitro *and *in vivo*.

## Methods

### Cell lines, tissues and reagents

The tissues used in our study belong to a set of clinical specimen already described [26].

Tumoral regression evaluation took into account measurements of the target lesions according to WHO recommendations for the evaluation of cancer treatment in solid tumors. Using computed tomography scanning, metastatic tumor size was estimated from bidimensional measurements (the product of the longest diameter and the longest perpendicular diameter) before and after every four or six cycles of chemotherapy to calculate the percent change from baseline. The study was approved by our local ethical committee; all participating patients were informed about the study and had to provide signed, written, informed consent before enrollment.

SW480 human colon adenocarcinoma was purchased from ATCC and was maintained in RPMI 1640 medium supplemented with 10% fetal bovine serum (FBS) and 2 mM L-glutamine. SN38 was kindly provided by Sanofi-Aventis. 5-Fluorouracile and oxaliplatin were kindly provided by Dr. F. Pinguet (CRLC Val d'Aurelle, France).

### cDNA arrays and analysis

Tumor and non tumor colon samples have been collected following a standardized procedure in order to obtain high quality RNA [27].

First strand cDNA synthesis was performed using a T7-linked oligo-dT primer and was followed by second strand synthesis. Labeled cRNA probes were then generated by reverse transcription followed by *in vitro *transcription, incorporating biotin labels, as part of the standard Affymetrix protocol. For each sample, the probes were then hybridized to human genome U133 chips (Affymetrix Inc., Santa Clara, CA), corresponding to genes and expressed sequence tags. Probes were then scanned, and signal intensity and the detection call for each transcript was determined using MAS 5.0 Software (Affymetrix Inc.). Inter-array normalization was performed using a set of internal standard genes for the determination of a scaling factor.

### RNA preparation and Q-PCR

All tissue samples were maintained at -196°C (liquid nitrogen) until RNA extraction. Tissue samples were then disrupted directly in a lysis buffer using Mixer Mill^® ^MM 300 (Qiagen, Valencia, CA). Total RNA was isolated using the RNeasy^® ^mini Kit (Qiagen), and subjected to additional DNAse digestion (Qiagen). RNA quantity was determined by UV spectroscopy. RNA purity and integrity were assessed using Agilent RNA 6000 Micro LabChip^® ^Kit (Agilent Technologies, Palo Alto, CA).

Random primers and dNTP used for the first-strand cDNA synthesis were purchased from Invitrogen. For real-time PCR analysis, LightCycler FastStart DNA Master SYBR Green kit (Roche Molecular Biochemicals, Mannheim, Germany) and the Light Cycler from Roche Diagnostics were used. Primer sets for hypoxanthine phosphorybosyl transferase (HPRT) and PSAT1 were taken from published sequences and are available upon request. The integrity of the PCR product reaction was verified by melting curve analysis. Real-time PCR values were determined by reference to a standard curve that was generated by Real-time PCR amplification of serially diluted cDNA using PSAT1 and HPRT primers. Values obtained for levels of PSAT1 were normalized to the levels of HPRT mRNA.

### Ectopic PSAT1 expression in SW480-FlpIn cells

PSAT1-expressing cells were generated with the Flp-In system (Invitrogen, San Diego, CA). SW480 adenocarcinoma-FlpIn host cell line was generated according to the instructions in the Flp-In System manual (Invitrogen). pcDNA5/FRT/PSAT1 was generated by inserting the PSAT1 open reading frame (from RZPD) into the pcDNA5/FRT/V5 vector (Invitrogen). PSAT1 was inserted into the genomic recombination site by FLP recombinase enzyme giving the SW480-PSAT1 cell line. Transfection of an empty vector into Flp-In site gave the SW480-FlpIn cell line. The stable cell lines were selected for hygromycin B resistance and cultured in RPMI 1640 (SW480) with 10% FBS and 50 μg/mL hygromycin B (Invitrogen). Single colonies were picked and expanded in selection medium. Vector inserts and the genomic DNA context in the selected clones were sequenced to verify the PSAT1 sequence and proper integration into the genetic locus.

### Western blot analyses

Non-transfected cells and stably transfected PSAT1 cells were lysed in SDS buffer (bromophenol blue, 5% β-mercaptoethanol, 2% SDS, 10% glycerol, 62.5 mM Tris-HCl pH 6.8). Extracts were sonicated and boiled for 5 min, and loaded and separated by SDS-PAGE (10%). Proteins were electrotransferred onto a polyvinylidene difluoride membrane (Amersham Pharmacia Biotech, Uppsala, AB, Sweden). Primary antibodies were a chicken polyclonal anti-human PSAT1 (M-49; GenWay Biotech, San Diego, CA, USA), a mouse monoclonal anti-Tag V5 (Invitrogen). Secondary antibodies were a horseradish peroxidase-conjugated goat anti-chicken and a horseradish peroxidase-conjugated anti-mouse (Sigma, St. Louis, MO). The proteins were detected by enhanced chemolumonescence (ECL) by using the ECL detection system from Amersham Pharmacia Biotech.

### Proliferation assay

Cells (5 × 10^4^) were seeded into six-well plates in triplicate. Six identical plates were seeded for each counting experiment. Every 24 hours after the initial seed, the cell number in one of the seven plates was determined.

### Mice and xenografting

Female athymic nu/nu mice were purchased from Harlan France and used at 6-8 weeks of age. For dualxenografts on the same mouse, 3 × 10^6 ^cells of each line were injected subcutaneously into the left flank for SW480-FlpIn and right flank for SW480-PSAT1. Tumors were detected by palpation and measured periodically with calipers. Experimental procedures and handling were performed in a laminar flow hood. Mice were euthanized when the tumor volume reached 200 mm^3^. All experiments and procedures were carried out under an animal protocol approved by the French departmental direction of veterinaries services. CG and BR are authorized under the numbers A34.142 and A34.220.

### Cytotoxic assay

Growth inhibition (IC_50_) assays were performed using the sulforhodamine B assay as already described [28]. Briefly, cells were seeded in 96-well plates (2000 cells per well) in complete medium. After a 48-hour rest, drugs were applied in a dilution series, each concentration in triplicate: 5-FU from 0 to 8 μM; SN38 from 0 to 10 nM; Oxaliplatin from 0 to 3 μM. After 72 hours, cells were fixed by adding trichloroacetic acid solution to a final concentration of 10% and stained with a 0.4% sulforhodamine B solution in 1% acetic acid (Sigma). The protein-bound dye was extracted with 10 mM Tris-HCl for determination of absorbance at 570 nm in an MRX plate reader (Dynex Inc., Virginia, USA). The IC_50 _values were determined graphically from the cytotoxicity curves. Each experiment was performed three times.

### Cell cycle analysis

The cells were seeded in 25 cm^2 ^flasks (4.5 × 10^4 ^cells/flask). After a 72-hour rest, the cells were treated for 72 hours with either 2 μM oxaliplatin, 20 nM SN38, or 10 μM 5-FU; or for 48 hours, with 1, 2, 4, and 8 μM oxaliplatin. One million cells were pelleted, washed with PBS, fixed in 75% ethanol, treated with 100 μg/mL of RNAse (Boehringer) and stained with 40 μg/mL of propidium iodide. Analyses were done on a FACScan flow cytometer (Becton Dickinson, Franklin Lakes, NJ, USA). The cells were gated on a dot plot display of forward scatter versus side scatter to extract aggregates. Cell cycle populations were quantified using WinMDI2.8 histogram analysis software (Phoenix Flow Systems, San Diego, CA).

### Apoptosis determination

Cells were seeded in six-well plates (3 × 10^4 ^cells/well). After a 72-hour rest, cells were treated 72 hours with either 2 μM oxaliplatin, 20 nM SN38, or 10 μM 5-FU. The cells (1 × 10^6^) and corresponding supernatants were labeled using the Annexin V-FLUOS Staining Kit (Roche Molecular Biochemicals, Mannheim, Germany) and propidium iodide (Sigma). Analyses were done on a FACScan flow cytometer (Becton Dickinson). Annexine V-FLUOS positive and PI negative cells were quantified using WinMDI histogram analysis software. For each analysis, the apoptosis percentage from the control cells was subtracted.

### Immunofluorescence microscopy

The cells were plated in culture dishes containing 12 mm glass coverslips. One day after oxaliplatin treatment (10 μM), cells on the coverslip were fixed in cold methanol then gradually rehydrated with PBS. The cells were incubated with an anti-tubulin monoclonal antibody (Sigma). Secondary antibody was a rhodamine conjugated anti-mouse. DAPI was used to stain the nucleus. Stained cells were mounted in Moviol, and images were recorded using a 63XNA objective on a Leica inverted microscope.

### Statistical analysis

To compare PSAT1 RNA expression between the 3 different tissues (NC, TC and HM) the Kruskal-Wallis test was used. Differences were considered significant when *p *≤ 0.05. The Pearson's test was used to calculate the correlation coefficient.

## Competing interests

The author(s) declare that they have no competing interests.

## Authors' contributions

NV performed the major part of experiments and drafted the manuscript. VC performed cloning and contributed to mRNA analysis and drug treatment analysis. CBM performed statistical analysis. VD contributed to cell proliferation analysis and cell preparation for mice experiments. NB performed cell staining and morphology observation. BR performed mice experiments. CF performed transcriptome analysis. EC contributed to transcriptome analysis. FM assisted with design of the study. CL assisted with design of the mitotic catastrophe experiment. PM assisted with design of the study and with critical examination of the manuscript. MDR provided affymetrix mRNA analysis and assisted with design of the study and with critical examination of the manuscript. CG coordinated the study, assisted with the design of experiments and drafted the manuscript. All authors read and approved the final manuscript.
